# Fire Behavior of Thermally Thin Materials in Cone Calorimeter

**DOI:** 10.3390/polym13081297

**Published:** 2021-04-15

**Authors:** Marouane El Gazi, Rodolphe Sonnier, Stéphane Giraud, Marcos Batistella, Shantanu Basak, Loïc Dumazert, Raymond Hajj, Roland El Hage

**Affiliations:** 1 Polymers Composites and Hybrids (PCH)—IMT Mines Ales, 30319 Ales, France; marouaneelgazi95@gmail.com (M.E.G.); marcos.batistella@mines-ales.fr (M.B.); loic.dumazert@mines-ales.fr (L.D.); raymond.hajj@hotmail.com (R.H.); 2 ENSAIT, GEMTEX–Laboratoire de Génie et Matériaux Textiles, 59000 Lille, France; stephane.giraud@ensait.fr; 3 Central Institute for Research on Cotton Technology, Adenwala Road, Matunga, Mumbai 400019, India; shantanubasak@gmail.com; 4 PR2N/LCPM, Faculty of Sciences, Lebanese University, Campus Fanar, Fanar P.O.B. 90656, Lebanon; roland_hag@ul.edu.lb

**Keywords:** flammability, cone calorimeter, thermally thin materials, textiles

## Abstract

In this study, a representative set of thermally thin materials including various lignocellulosic and synthetic fabrics, dense wood, and polypropylene sheets were tested using a cone calorimeter at different heat fluxes. Time-to-ignition, critical heat flux, and peak of heat release rate (pHRR) were the main parameters considered. It appears that the flammability is firstly monitored by the sample weight. Especially, while the burning rate of thermally-thin materials does never reach a steady state in cone calorimeter, their pHRR appears to be mainly driven by the fire load (i.e., the product of sample weight and effective heat of combustion) with no or negligible influence of textile structure. A simple phenomenological model was proposed to calculate the pHRR taking into account only three parameters, namely heat flux, sample weight, and effective heat of combustion. The model allows predicting easily the peak of heat release rate, which is often considered as the main single property informing about the fire hazard. It also allows drawing some conclusions about the flame retardant strategies to reduce the pHRR..

## 1. Introduction

Thermally thin materials are defined as materials for which there is no significant heat gradient throughout their thickness. In other words, the whole volume is heated at the same temperature at all times. On the contrary, the heat gradient is significant in thermally thick materials. The flammability of thermally thin materials, such as textiles, is of great concern. Indeed, they are present in buildings such as in curtains and upholstered furniture and can greatly contribute to residential fires [1,2]. Moreover, some textiles are subject to specific fire issues (nightwear, business uniform) [3,4]. Therefore, many studies have been carried out in the past to better understand the flammability of thermally thin materials and to provide efficient flame retardant solutions [5].

A cone calorimeter is probably the apparatus the most used by the scientific community working on the flammability of materials. It provides quantitative data on various properties, including ignitability, heat release, gas, and smoke release. A comprehensive review can be found to make the best use of this device [6]. It has been highlighted that the choice of setup conditions influences significantly the fire performances and these conditions must be chosen carefully [7]. This is especially the case for thermally thin materials, such as textiles or films. Indeed, such materials are semi-transparent and may not absorb the entire applied heat flux. Moreover, they can distort during burning and, therefore, the exposed surface area is not constant anymore, making the calculation of the heat release rate (HRR) false. Some researchers have already investigated the effect of setup (substrate, edge effects, grid) on fire performances of textiles in order to limit these phenomena [8,9]. However, despite these disturbances, the cone calorimeter still remains a useful tool even for such materials and has often been used to characterize the fire performances of various textiles [9,10,11,12,13]. Nevertheless, the role of the textile structure on flammability properties is not clear. Hernandez et al. [13] have shown that flammability of polypropylene (PP) materials in the cone calorimeter is mainly driven by their area density and not by the textile structure. However, PP melts at 160 °C, which is a temperature much lower than its decomposition temperature and, therefore, the textile structure disappears prior to the decomposition and, thus, has no influence on fire behavior.

Time-to-ignition (TTI) has been extensively studied and some models have been proposed for its prediction under various experimental conditions, such as flat or cylindrical geometries, thermally thick or thin materials, auto-ignition or piloted ignition, constant or variable heat flux [14,15,16,17,18,19,20,21]. Equations (1) and (2) are quite often used to predict the time-to-ignition for, respectively, thermally thick and thermally thin materials under constant heat flux [21]:

(1) $$TTI=\frac{\pi }{4}k\rho c{\left[\frac{T_{ig}-T_{0}}{\varepsilon q_{ext}^{”}-CHF}\right]}^{2}$$

(2) $$TTI=l\rho c\frac{T_{ig}-T_{0}}{\varepsilon q_{ext}^{”}-CHF}$$

With *k* being the heat conductivity, *ρ* the density, *c* the specific heat, *l* the sample thickness, *T_ig_* the temperature at ignition, *T*_0_ the room temperature, $q_{ext}^{"}$ the heat flux, and *CHF* the critical heat flux.

Models have also been proposed to account for steady burning in the case of thermally thick materials [19,20]. Of course, the steady state is not always reached even for thermally thick materials. Indeed, some phenomena, such as char accumulation, may reduce the heat transfer leading to a continuously decreasing heat release rate (HRR). Stoliarov et al. have proposed a model to predict the burning rate of charring polymers [22]. 

As explained by Schartel and Hull [6], thermally thin materials are “characterized by a sharp peak in HRR, since the whole sample is pyrolyzed at the same time. In this case, the pHRR (peak of heat release rate) becomes dependent on their total fire load”. It means that these materials do not show any steady burning rate and pHRR is only dependent on the sample weight at fixed conditions. Indeed, fire load is the product of the fuel mass and the effective heat of combustion. In a cone calorimeter, which is a well-ventilated test, effective heat of combustion is usually close to the heat of complete combustion. The latter can easily be calculated using Huggett’s relation [23] if the material burns completely or can be measured using pyrolysis combustion flow calorimeter. Nevertheless, to the best of our knowledge, there has been no attempt to check if pHRR of thermally thin materials may be easily calculated only from their fire load. Thompson and Apostolakis have attempted to predict the pHRR of upholstered furniture containing a fabric using a response surface analysis and using several parameters, including thermal inertia, heat of combustion, and heat flux, but they considered a much more limited set of materials and test conditions [2].

This work has two respective objectives. First, it aims to investigate the role of the structure of lignocellulosic (not thermoplastic) fabrics on its flammability in a cone calorimeter. Second, it aims to check whether the fire load drives pHRR of thermally thin materials and to propose a simplistic phenomenological model able to predict it.

## 2. Materials and Methods

**Figure 1:**
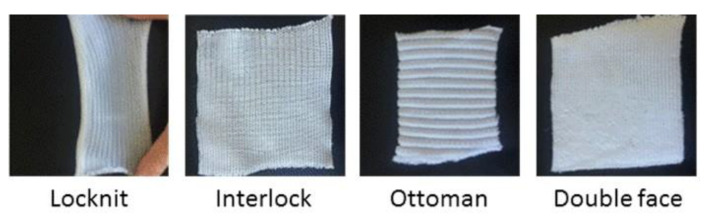
Examples of cotton knitted fabrics.

The flammability of several series of materials has been investigated. These materials come from different projects, and the test conditions may slightly differ:

- Ten cotton woven fabrics with an area density ranging from 80 to 270 g/m^2^. The first eight fabrics (called T1 to T8) are not flame retarded. The other fabrics (called F1 and F3) are derived from T8 sample. T8 is flame retarded using a flame retardant (FR) system based on ammonium polyphosphate (APP) (10–12 µm; Phosphorus and nitrogen contents are respectively 31–32 wt% and 14–15 wt%) provided by Focus Química (São Paulo, Brazil) and sodium montmorillonite (Na-Mt) under the trade name Cloisite^®^ Na+ (Southern Clay Products, Gonzales, TX, USA).
- Two flax fabrics (area density of 200 g/m^2^), respectively unmodified and flame retarded with a phosphorus additive (vinyl phosphonic acid) grafted using irradiation [24].
- Three jute fabrics of plain-woven material (area density of 180 g/m^2^) unmodified and flame retarded with Rochelle salt (Potassium sodium tartrate) and borax (sodium borate), respectively.
- Four cotton knitted fabrics with different structures: Locknit, Double Face, Interlock, and Ottoman (Figure 1). More details can be found in Hernandez et al. [13].
- Four flax knitted fabrics with the same structures.
- Five PP knitted fabrics with the same structures (studied in a previous work [13]).
- Four PP sheets with a thickness ranging from 1 to 6 mm (studied in a previous work [13]).
- Two aramid-based fabrics (Twill). The exact composition of these two fabrics is unknown. Note that for these two fabrics, the external heat fluxes ranged from 20 to 50 kW/m^2^.
- One thin (1 mm-thick) sheet of low-density wood, namely balsa (density < 150 g/m^3^). Note that no grid was used in this case.

Main properties of these samples are listed in Table 1. Thirty-five different materials were tested in most cases at several heat fluxes. PP fabrics have already been studied [13]. In the present study, we have chosen to test additional lignocellulosic fabrics because these fabrics do not melt and then their structure is maintained during a longer period in the cone calorimeter. Moreover, effective heat of combustion (EHC) of PP is close to 40 kJ/g versus around 12 kJ/g for lignocellulosic materials. This allows us to discriminate the influence of EHC and initial mass on flammability as discussed later. Finally, some other materials (wood sheet, FR fabrics, and complex aramid-based fabrics) were added to extend the range of thermally thin materials, ensuring that our findings concern a large set of materials.

**Figure 2:**
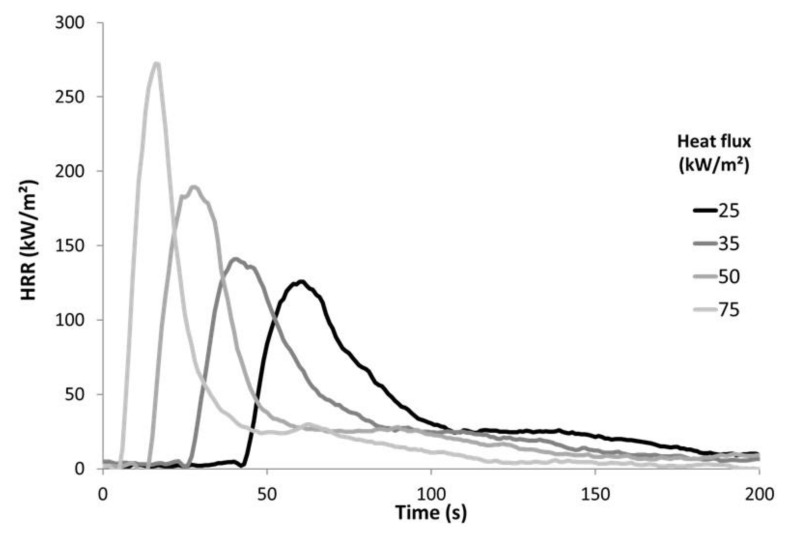
HRR curves for jute fabric.

The flammability was investigated using a cone calorimeter (Fire Testing Technology, East Grinstead, UK) according to the ISO 5660 standard. We have chosen to follow the same procedure as in our previous work on PP textiles flammability [13]. A horizontal sample sheet of 100 × 100 mm^2^ was placed at 2.5 cm below a conic heater. Typically, samples were exposed to various heat fluxes (25, 35, 50, and 75 kW/m^2^) in well-ventilated conditions (air rate 24 L/s) in the presence of a spark igniter to force the ignition. To avoid the distortion of samples during the test (see for example Locknit structure on Figure 1), a grid was used as suggested by Tata et al. [9]. The samples were isolated by rock wool as in standard tests. The edges as well as the bottom of the samples were covered by aluminum foil as usual. Due to the fast burning of these materials, the measurement time step was reduced to 1 s. Figure 2 shows the HRR curves for jute fabric as an example.

HRR was determined according to oxygen depletion using Huggett’s relation [23]. Altogether, 8–12 samples were tested for each formulation (only four for cotton fabrics). Mean values are calculated for each sample and each heat flux, and the coefficients of determination of the curves 1/TTI (or pHRR) = f($q_{ext}^{"}$*)* are calculated from these mean values. The coefficients of determination are generally high, but we have chosen to maintain in this study even the materials for which they are less satisfying. Despite that the lignocellulosic materials are able to char, char contents are not considered in this study. Indeed, after flame out, thermo-oxidation of char occurs. However, the duration between the flame out and the end of the test was not monitored. Moreover, the mass loss rate curve is too noisy due to the fact that very thin materials burn very fast. Furthermore, the flame retarded textiles exhibit higher char contents at the end of the test, evidencing the efficiency of the FR systems.

EHC was measured for each test and mean values were calculated. EHC remains constant whatever the applied heat flux, except for aramid-based fabrics. For these fabrics, at low heat flux, a fraction of materials is charring but the char is not thermo-oxidized after flame out, probably because the temperature is too low. Except these fabrics, total heat release (THR) may be easily calculated as the product of EHC and mass loss rate (which is equal to initial mass in most cases).

This work is focused on three main properties, namely TTI, critical heat flux (CHF), and pHRR. It should be noted that TTI and pHRR are among the most meaningful parameters to study the flammability but also to establish some correlations between full-scale and bench-scale tests. For example, empirical relations including TTI and pHRR were proposed to predict the flashover of surface linings [25,26].

## 3. Results

It is well known that the thickness has a strong influence on the flammability for thermally thin materials [7]. In order to compare dense materials and textiles, a more convenient way is to consider the area density or the sample weight when the exposed surface is constant. In the case of PP materials, Hernandez et al. [13] showed that not only the flammability properties but also their dependence on heat flux are impacted by these parameters.

### 3.1. Thermal Penetration

Most of materials tested in this work are thermally thin but some of them may exhibit an intermediate behavior. This is particularly the case of thick PP sheets, as shown in Hernandez et al. [13].

Considering Equations (1) and (2), the TTI dependence on heat flux is different for thermally thick and thermally thin materials. One method to check the thermal behavior of materials is to draw 1/TTI or 1/√TTI versus heat flux $q_{ext}^{"}$. Nevertheless, in most cases, both curves 1/TTI = f($q_{ext}^{"}$) and 1/√TTI = f($q_{ext}^{"}$) are well linear. However, in this article, the coefficient of determination R^2^ is high in both cases. It is, thus, not possible to identify the thermal behavior using this method.

Another method consists in calculating the thermal penetration. A material is thermally thin when the temperature is the same throughout its entire volume, i.e., there is no significant temperature gradient between its upper and lower surface. In this case, the depth of thermal penetration δ is higher than the physical thickness of the sample [21]. δ is then a critical thickness calculated as follows:

(3) $$\delta \approx \sqrt{\alpha .TTI}$$

where δ is thermal penetration (mm), α is thermal diffusivity (mm^2^/s), and TTI is time to ignition (s).

The thermal penetration of PP sheets was calculated considering that thermal diffusivity of PP is 0.89 × 10^−7^ mm^2^/s [27]. The values are reported in Table 2. From these data, it is clear that 6 and 4 mm thick PP sheets cannot be considered as thermally thin, whatever the heat flux. On the contrary, 1 mm thick sheet is thermally thin. For an intermediate PP sheet (2 mm thickness), the thermal behavior depends on heat flux. At low heat flux, the sheet is considered as thermally thin. At high heat flux, the thermal penetration becomes lower than the sample thickness. Nevertheless, we will include all PP data in the following. Note that Equation (3) is only a rough criterion to discriminate between both thermal regimes. For example, Lyon et al. consider that Equation (1) for thermally thick materials is suitable when the thickness is higher than 2√(α.TTI) [28].

### 3.2. Time-to-Ignition

For thermally thin dense materials Equation (2), TTI is dependent on thickness *l*ρ (i.e., the product of thickness and density, which increases with sample weight). Other influent material parameters are specific heat *c* (the range of this parameter is quite limited for polymers) and thermal stability (or more exactly temperature of ignition *T_ig_*).

**Figure 3:**
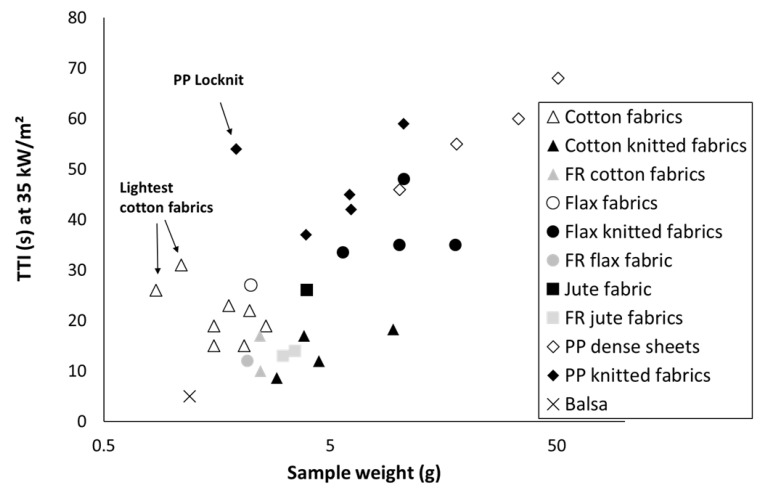
Time-to-ignition (TTI) at 35 kW/m^2^ versus sample weight.

Table 3 summarizes all recorded data about time-to-ignition for materials studied in the present article. Obviously, TTI decreases when heat flux increases. Differences in TTI are more obvious at low heat flux. As an example, Figure 3 plots the time-to-ignition at 35 kW/m^2^. TTI roughly increases when sample weight increases. Some exceptions correspond to very light samples (as the lightest cotton fabrics T5 and T7) and Locknit PP. Hernandez et al. have noted that data are less reliable for this fabric due to dimensional instability during the test, even when the grid is used [13]. For similar sample weight, TTI is systematically higher for flax than for cotton. This may be ascribed to different parameters as the thread size or the presence of fibrils on sample surface. Flame retardancy of cotton or flax fabrics lowers significantly the time-to-ignition. This is not surprising considering the role of phosphorus flame retardants, which promote charring by dehydrating polymer chains but reduce their thermal stability. A similar effect is also observed for FR jute fabrics even if FRs are not based on phosphorus.

**Figure 4:**
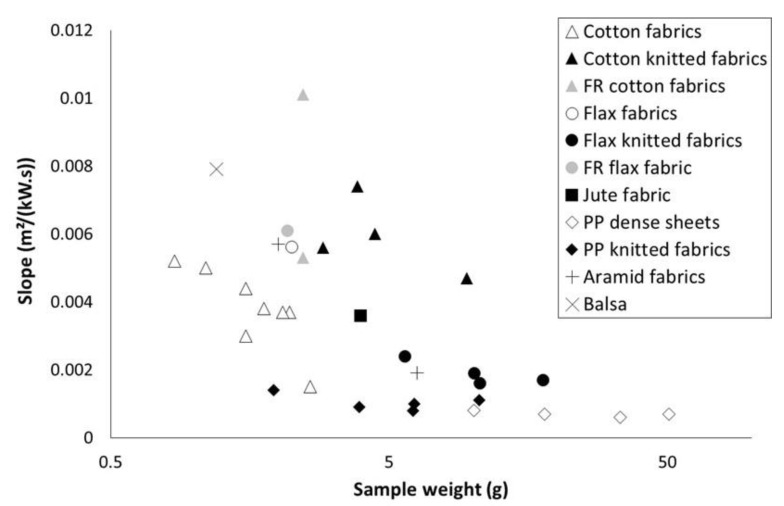
Slope of $\frac{1}{TTI}=f\left(q_{ext}^{\text{”}}\right)$ curves versus sample weight.

The slope of the curve 1/TTI = f($q_{ext}^{"}$) is plotted versus sample weight in Figure 4. According to Equation (2), the lower the sample weight is, the higher is the slope. This trend is confirmed in Figure 4. For a same series (for example cotton fabrics), the slope tends to increase when sample weight decreases. When sample weight exceeds 5 g, the slope appears to be almost insensitive to weight. Nevertheless, other parameters should be taken into account. Flame retardancy may lead to significant slope changes (please compare data for the two FR cotton fabrics). Especially, the comparison between cotton fabrics and cotton knitted fabrics may show that the structure has a significant effect on the slope (beyond its effect on area density). Nevertheless, while the cotton in both series is not the same, it is difficult to conclude about this effect. Differences are also observed between flax and cotton fabrics with similar weight. Slope for PP materials is very low, even when the sample weight is low. This may be ascribed to the thermal stability of PP. Indeed, in pyrolysis combustion flow calorimetry, temperature at pHRR is usually as high as 480 °C for PP, versus 360 °C for lignocellulosic materials.

Critical Heat Flux (CHF) is another material property listed in Table 3. CHF was calculated by extrapolating the curve 1/TTI = f($q_{ext}^{"}$). Various CHF values can be found elsewhere [29]. CHF of various (lignocellulosic and synthetic) fabrics was found in the range 5–20 kW/m^2^ by Babrauskas and Parker and Nazaré et al. [8,11]. Despite some variations (T1), CHF is around 20 kW/m^2^ for cotton as well as for flax and jute fabrics. FR lignocellulosic fabrics exhibit similar CHF values. These values are slightly higher than the values calculated from data of Nazaré et al. (i.e., 10 and 17 kW/m^2^ for heavy and light cotton respectively) [11]. CHF for PP materials (fabrics and dense sheets) is close to 12 kW/m^2^ in good agreement with Lyon and Quintiere [29]. CHF for aramid-based fabrics appears quite low (5 kW/m^2^) while aramid is considered as heat-resistant polymer. Nevertheless, the exact composition of these fabrics is not known and flammability may be enhanced by other components. CHF for balsa was found to be 10.6 kW/m^2^, close to the value obtained by Mikkola et al. [21] for thermally thin spruce (12 kW/m^2^).

According to the present results, it should be assumed that CHF depends mainly on the material nature and is not greatly influenced neither by area density nor by textile structure. This conclusion is confirmed for materials for which the structure is not collapsed too soon due to melting, as flax or cotton. For dense PP sheets, we assume that the tendency is not reliable enough to conclude about an increase of CHF with the area density.

### 3.3. Peak of Heat Release Rate

**Figure 5:**
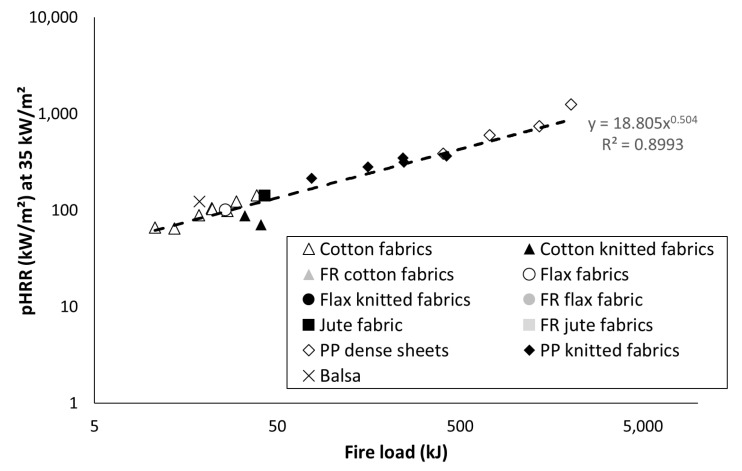
Peak of heat release rate (pHRR) at 35 kW/m^2^ versus fire load.

As already stated, peak of heat release rate is considered as one of the main flammability parameters to estimate the fire hazard. PHRR values are listed in Table 4 for all studied materials. The main objective of this work is to assess the correlation between pHRR and fire load. Figure 5 shows this correlation at 35 kW/m^2^ as an example. Despite the great variability of tested materials, a rough correlation can be observed: pHRR increases when fire load increases, according to a power law. Note that such relation is also observed for other heat fluxes (data not shown). Most of materials, especially lignocellulosic ones, exhibit a pHRR lower than 200 kW/m^2^. PP materials exhibit much higher pHRR even for the lightest samples. This can be assigned to its higher effective heat of combustion (close to 40 kJ/g versus 10–15 kJ/g for lignocellulosic materials).

From these results, it can be assumed that pHRR is not influenced by the textile structure, even for lignocellulosic materials, which do not melt during heating. Of course, this observation is valid only for the present test.

Comparing FR cotton (F1 and F3) and cotton fabric (T1) for similar sample weight, the decrease in pHRR is related to a decrease in EHC. Similar conclusion can be drawn from the comparison between non flame-retarded and FR flax fabrics.

**Figure 6:**
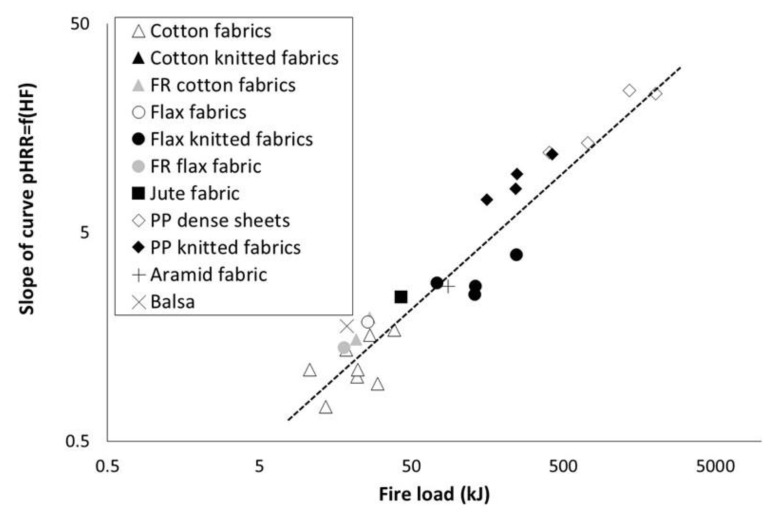
Slope of the curve pHRR = f(heat flux) versus fire load (dotted line is only shown for eye guideline).

In all cases, pHRR increases when heat flux increases. Moreover, this tendency is linear as already observed by Nazaré et al. [11]. Slope of the curve pHRR = f($q_{ext}^{"}$) was calculated and listed in Table 4. Values are plotted versus fire load in Figure 6 (except for Locknit PP knitted fabric due to a too low coefficient of determination). A rough correlation is observed between this slope and the fire load, according to a power law. It means that the higher the fire load is, the more sensitive is the pHRR to the heat flux.

Once again, there is no significant influence of textile structure on the slope value. FR fabrics (flax and cotton) exhibit a lower sensitivity to heat flux (lower slope value) due to a decrease in fire load (because of a lower EHC).

All these observations confirm that the pHRR of thermally thin materials greatly depends on the fire load only.

### 3.4. Phenomenological Modelling

The objective of this part is to propose a phenomenological model allowing predicting the pHRR of thermally thin materials from a set of three parameters, namely, heat flux, sample weight, and effective heat of combustion. Such modelling would be helpful because it does not require the knowledge of many material properties, which are sometimes difficult to measure, especially for textiles.

We have already noted that some materials are not thermally thin. Nevertheless, we have chosen to keep these data to elaborate the model discussed below.

Peak of HRR and peak of mass loss rate (pMLR) are simply related according to Equation (4):

(4) pHRR=pMLR×EHC

Effective heat of combustion depends on the nature of materials but not on its weight. Usually it does not depend greatly on heat flux. In cone calorimeter conditions (i.e., well-ventilated conditions), EHC is often close to the heat of complete combustion.

**Figure 7:**
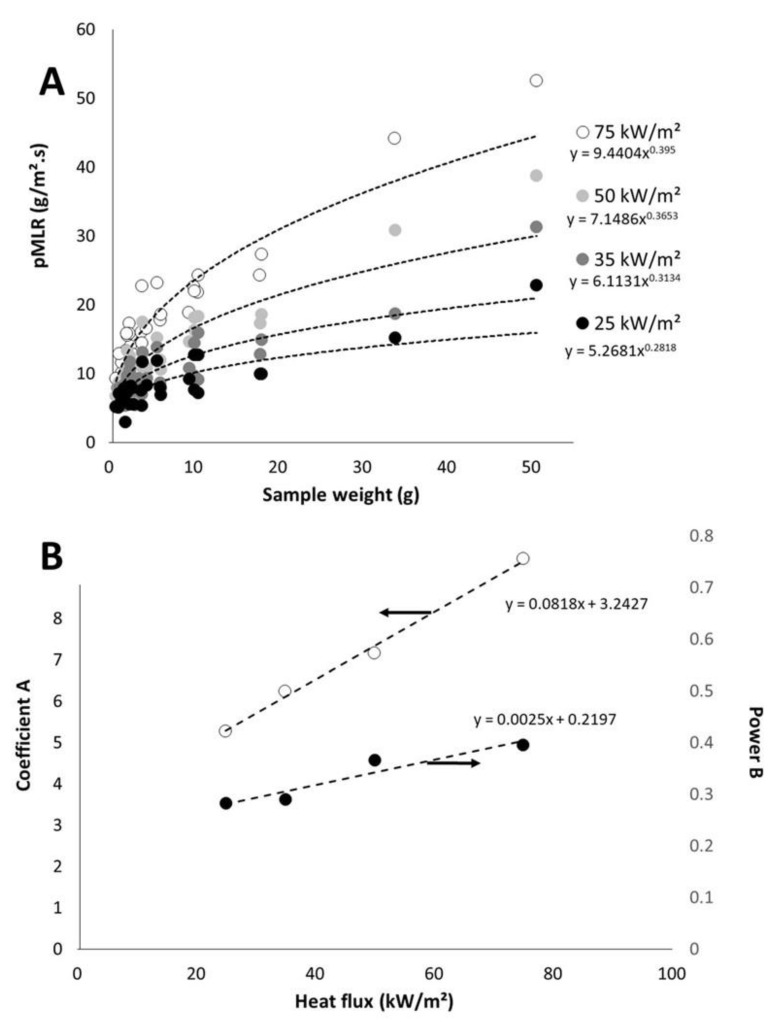
Peak of mass loss rate versus sample weight for different heat fluxes (**A**); Values for coefficient A and power B versus heat flux (**B**).

On the contrary, mass loss rate depends on heat flux. Figure 7A plots the pMLR (i.e., the ratio between pHRR and EHC) versus sample weight for different heat fluxes. Even if the coefficient of determination is not high, we will consider that pMLR depends on the sample weight *w* according to the Equation (5).

(5) $$pMLR=A\times w^{B}$$

Note that A and B increase both when heat flux increases (Figure 7B). A may be defined as the pMLR of a 1 g-sample. The value for B is lower than 1, whatever the heat flux. It means that pMLR increases slower and slower when the sample weight increases. In other words, when the sample weight becomes high, material behavior turns from thermally thin to thermally thick and pMLR does not increase anymore. This is in agreement with the expected behavior of a thermally thick material.

Plotting A and B versus heat flux leads to Figure 7B. Both coefficients change linearly with heat flux (at least in the range 25–75 kW/m^2^) according to Equations (6) and (7) respectively:

(6) $$A=0.0818\times q_{ext}^{”}+3.2427$$

(7) $$B=0.0025\times q_{ext}^{”}+0.2197$$

Therefore, pHRR can be easily calculated according to the following procedure. The knowledge of heat flux allows to calculate A and B. Then pMLR can be calculated for a sample of a known weight. PHRR is finally deduced as the product of pMLR and EHC. In the case of most materials, in cone calorimeter, heat of complete combustion could be used instead of EHC. In the present study, EHC was measured during the cone calorimeter but results would be similar if the heat of complete combustion measured in pyrolysis-combustion flow calorimetry was considered instead of EHC.

**Figure 8:**
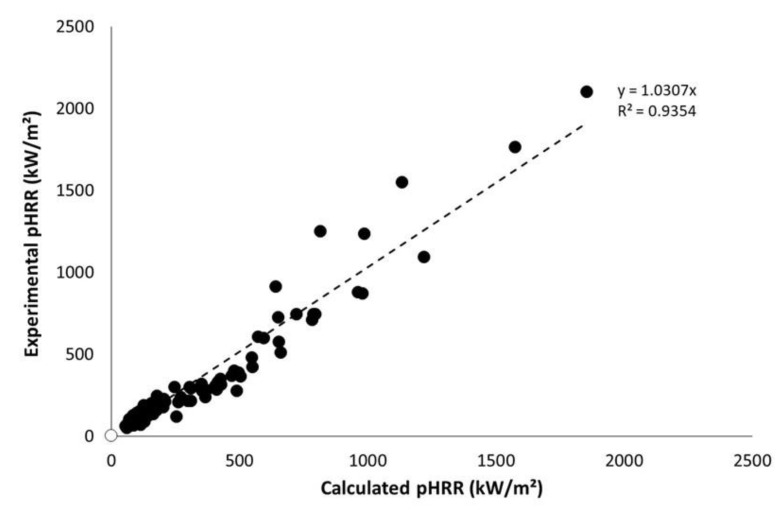
Experimental versus calculated pHRR for all materials tested in this study.

Figure 8 shows the calculated pHRR versus experimental ones according to the procedure proposed above. The coefficient of determination is acceptable (R^2^ > 0.93) considering the type of samples. It is usually considered that uncertainties on pHRR are close to 15% in cone calorimeter. Nevertheless, this is true mainly for flame-retarded materials exhibiting thermally thick behavior. Uncertainties are often much higher for thermally thin materials. For highly flammable materials such as PP without flame retardant, pHRR may also vary widely.

Note that these data include a large set of samples, in terms of weight, type of samples (sheets, dense wood, fabrics), and nature of materials. Indeed, materials range from lignocellulosic ones with low thermal stability and heat of combustion to synthetic polymers with higher thermal stability and very high heat of combustion (and some aramid-based fabrics but their exact composition is unknown).

It is also noteworthy that pHRR is quite well predicted for non-thermally thin materials considered in this study, i.e., for the thickest PP sheets (exhibiting the highest pHRR). It means that the behavior of these samples remains (more or less) thermally thin.

**Figure 9:**
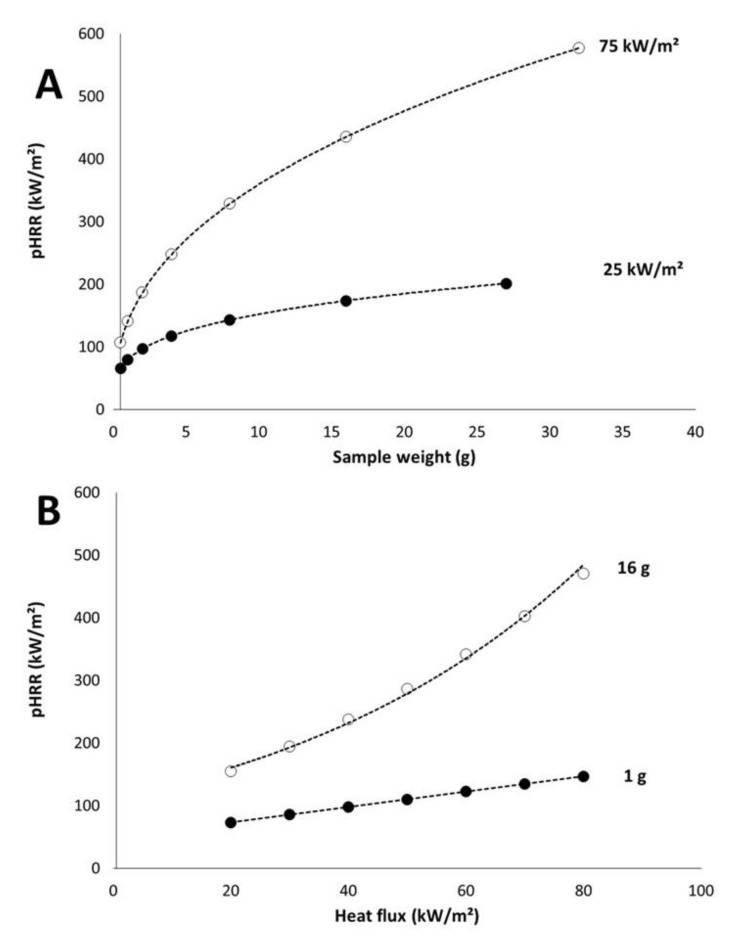
Calculated pHRR versus sample weight (**A**) and heat flux (**B**) (effective heat of combustion (EHC) = 15 kJ/g).

Figure 9A shows the modelled pHRR versus sample weight for two heat fluxes and versus heat flux for two sample weights. EHC was fixed to 15 kJ/g and pHRR increases when sample weight increases. Nevertheless, the increase is quite moderate at low heat flux. Increasing the weight from 0.5 to 32 g (this weight is certainly beyond the limit for a thermally thin behavior) leads to an increase of pHRR from 65 to 211 kW/m^2^. For sample weight ranging from 3 to 10 g, pHRR is in a narrow range: 100–150 kW/m^2^. At high heat flux, the increase is much more significant: from 106 to 577 kW/m^2^ when sample weight increases from 0.5 to 32 g.

Considering pHRR versus heat flux (Figure 9B), similarly the change is relatively limited for the lightest sample (1 g): pHRR increases from 73 to 146 kW/m^2^ when heat flux increases from 20 to 80 kW/m^2^. For the same range of heat flux, pHRR increases from 155 to 470 kW/m^2^ in the case of a sample weighing 16 g.

In their study on a limited set of upholstered furniture, Thompson and Apostolakis have also pointed out the influence of heat flux, heat of combustion, and sample weight (they consider the thermal inertia which is obviously related to sample weight) on pHRR [2]. These authors noted that sample weight has a lower influence than heat flux (ranging from 20 to 40 kW/m^2^) and heat of combustion. At these low to moderate heat fluxes, our own results confirm that the sample weight moderately influences the pHRR.

### 3.5. Some Comments about the Flame Retardancy of Thermally Thin Materials

The possibility to predict the pHRR of thermally thin materials using a very simplistic model considering only three parameters (namely sample weight, heat flux, and effective heat of combustion) is meaningful. Indeed, this model means that there is no significant thermal gradient in the material and the decomposition rate is limited only by the amount of fuels. This is the expected behavior of such materials.

The model confirms that the textile structure has no direct influence on pHRR in such a test. This conclusion may be extended to other sample types (like dense sheets). Nevertheless, the textile structure has an effect on area density and, thus, on sample weight and fire load. This effect can be considered as indirect.

Other comments can be formulated on the strategies to improve the flame retardancy of thermally thin materials as far as the flame retardant strategies do not change the thermal behavior of the material (i.e., from thermally thin to thermally thick behavior). Flame inhibition makes combustion incomplete and decreases the effective heat of combustion. Therefore, this strategy appears to be efficient while the peak of heat release rate depends directly on this parameter.

Char promotion also tends to decrease the effective heat of combustion. Indeed, char is usually carbon-rich. Its structure can be often considered as close to C_5_H_2_ with a high heat of combustion (37.2 kJ/g) [30]. As far as this assumption is valid, for materials exhibiting initially a lower heat of combustion (as natural fibers), charring leads to a release of carbon-poor fuels with a reduced heat of combustion.

Nevertheless, charring also allows the accumulation of char on the top surface of the material, leading to a protective insulating char layer. This barrier effect is known to be much more efficient for thick materials, as already noticed by Schartel et al. [7]. For initially thermally thin materials, such a barrier should also change the behavior from thin to thick. A comparison between experimental pHRR and the predicted ones using the present model may be an indicator of the efficiency of this mode-of-action. Further investigations are needed to confirm this proposal.

Of course, if char promotion is very important, pHRR should be reduced not only by a decrease of EHC but also additionally by fuel shortage, i.e., the amount of pyrolyzed sample contributing to heat release is reduced. In this case, the sample weight in the model should be replaced by the amount of released fuel.

The release of water by hydrated minerals (as aluminum hydroxide (ATH) or magnesium hydroxide (MDH)) may also reduce effective heat of combustion. Nevertheless, this effect is quite limited and needs a high amount of flame retardants. Indeed, ATH and MDH release only 35 and 31 wt% of water, respectively.

Finally, strategies acting on the thermophysical properties as specific heat or heat conductivity but also on thermal stability should not be effective to reduce the pHRR, while these properties do not appear as influent parameters in the model.

## 4. Conclusions

An extended set of thermally thin materials was tested in cone calorimeter including cellulosic and lignocellulosic fabrics with different textile structures. The textile structure controls the area density and, therefore, the sample weight. However, even when the materials do not melt, there is no other influence of the structure on the flammability, especially on pHRR.

Ignition is mainly monitored by the sample weight. Time-to-ignition increases for heavier samples. A monotonic dependence of TTI on sample weight is observed. Critical heat flux was remarkably constant for cellulosic and lignocellulosic fabrics, close to 20 kW/m^2^, irrespective to the sample weight or its structure, confirming that it is an intrinsic material property. Some differences especially in terms of TTI can be found between cotton and flax and need further investigations.

Interestingly, peak of heat release rate can be calculated using only three parameters: heat flux, sample weight, and effective heat of combustion. More precisely, the peak of heat release rate increases when heat flux, sample weight, or effective heat of combustion increase. This model confirms that pHRR is proportional to fire load but also shows that the dependence on heat flux is irrespective of the material (i.e., depends only on sample weight).

As far as the model is concerned, strategies to improve flame retardancy of thermally thin materials can be inferred. Indeed, according to the model, the only intrinsic property contributing to the peak of heat release rate is the effective heat of combustion. This value ranges from few kJ/g (for fluorinated polymers for example) to 44 kJ/g for polyethylene if the combustion is complete. Flame inhibition directly affects the effective heat of combustion. Char promotion also lowers this value in most cases, because char is usually more carbon-rich than the starting material. Moreover, for charring materials, the sample weight should be probably replaced by the mass loss in the model.

## Figures and Tables

**Table 1:** Main characteristics of thermally thin materials considered in this work.

Sample	Area Density (g/m^2^)	Sample Weight (g)	Thickness (mm)	Textile Structure
Cotton Fabric (T1)	270	2.6	0.4	Plain Weave
Cotton Fabric (T2)	210	2.2	0.5	Plain Weave
Cotton Fabric (T3)	150	1.53	0.6	Plain Weave
Cotton Fabric (T4)	180	1.78	0.6	Plain Weave
Cotton Fabric (T5)	110	1.1	0.4	Plain Weave
Cotton Fabric (T6)	170	1.53	0.6	Plain Weave
Cotton Fabric (T7)	80	0.85	0.7	Plain Weave
Cotton Fabric (T8)	210	2.08	1.3	Plain Weave
FR T8 Cotton Fabric (F1)	250	2.46	0.5	Plain Weave
FR T8 Cotton Fabric (F3)	250	2.45	0.5	Plain Weave
Locknit Cotton Knit	200	2.9	1.2	Locknit
Interlock Cotton Knit	400	3.84	2.8	Interlock
Double Face Cotton Knit	500	4.46	2.5	Double Face
Ottoman Cotton Knit	900	9.5	4.8	Ottoman
Flax Fabric	200	2.24	0.4	Plain Weave
FR Flax Fabric	200	2.16	0.42	Plain Weave
Locknit Flax Knit	700	5.7	3.1	Locknit
Interlock Flax Knit	1100	10.6	3.2	Interlock
Double Face Flax Knit	1100	10.1	4.1	Double Face
Ottoman Flax Knit	1850	17.9	5	Ottoman
Jute Knit	380	3.95	2.1	Plain Weave
Jute Knit (Rochelle Salt)	/	3.5	2.1	Plain Weave
Jute Knit (Borax)	/	3.1	2.1	Plain Weave
Locknit PP Knit	218	1.9	1.3	Locknit
Interlock PP Knit	442	3.9	2.6	Interlock
Double Face PP Knit	690	6.1	3.3	Double Face
Interlock 2 Yarns PP Knit	698	6.2	3	Interlock
Ottoman PP Knit	1195	10.6	5.4	Ottoman
1 mm-Thick PP Sheet	1145	10.1	1	Sheet
2 mm-Thick PP Sheet	2048	18.1	2	Sheet
4 mm-Thick PP Sheet	3831	33.9	4	Sheet
6 mm-Thick PP Sheet	5729	50.6	6	Sheet
Balsa	/	1.2	1	Sheet
Thin Aramid Fabric	/	2	/	Twill
Thick Aramid Fabric	/	6.3	/	Twill

**Table 2:** Thermal penetration δ (in mm) of PP sheets.

Thickness (mm)	Heat Flux (kW/m^2^)			
	25	35	50	75
1	2.4	2.0	1.6	1.3
2	2.8	2.2	1.8	1.4
4	3.0	2.3	1.9	1.5
6	3.4	2.5	1.9	1.4

**Table 3:** Main data for ignition of thermally thin materials from cone calorimeter tests.

Sample	Area Density (g/m^2^)	Sample Weight (g)	25 kW/m^2^	35 kW/m^2^	50 kW/m^2^	75 kW/m^2^	Slope (m^2^/(kW.s))	R^2^	CHF (kW/m^2^)
Cotton Fabric (T1)	270	2.6	56.5	19	19	10	0.0015	0.91	8.6
Cotton Fabric (T2)	210	2.2	48.5	22	8	5	0.0037	0.98	20
Cotton Fabric (T3)	150	1.53	57	19	8	6	0.003	0.95	16.5
Cotton Fabric (T4)	180	1.78	71	23	10	5	0.0038	1	22.3
Cotton Fabric (T5)	110	1.1	65	31	7	4	0.005	0.97	23.8
Cotton Fabric (T6)	170	1.53	44	15	11	4	0.0044	0.95	22.2
Cotton Fabric (T7)	80	0.85	61	26	4	4	0.0052	0.76	19.4
Cotton Fabric (T8)	210	2.08	69	15	7	5	0.0037	0.96	17.4
FR T8 Cotton Fabric (F1)	250	2.46	28	10	2	2	0.0101	0.77	18.3
FR T8 Cotton Fabric (F3)	250	2.45	39	17	7	3.5	0.0053	0.99	22.2
Locknit Cotton Knit	200	2.9	23	8.7	6	3	0.0056	0.99	17.1
Interlock Cotton Knit	400	3.84	26.5	17	9	2.5	0.0074	0.91	25.5
Double Face Cotton Knit	500	4.46	35	12	7	3	0.006	0.98	22.1
Ottoman Cotton Knit	900	9.5	41	18.3	5.5	4	0.0047	0.94	19.6
Flax Fabric	200	2.24	78	27	11	4	0.0056	0.96	26.6
FR Flax Fabric	200	2.16	38	12	7.5	3	0.0061	0.97	22.2
Locknit Flax Knit	700	5.7	63	33.5	19	7.5	0.0024	0.96	21.5
Interlock Flax Knit	1100	10.6	92	48	21	11	0.0016	0.99	20.8
Double Face Flax Knit	1100	10.1	81	35	19	10	0.0019	0.99	19
Ottoman Flax Knit	1850	17.9	88	35	24	10	0.0017	0.97	20.7
Jute Knit	380	3.95	44	26	13	5	0.0036	0.96	22.9
Jute Knit (Rochelle Salt)	/	3.5	/	14	/	/	/	/	/
Jute Knit (Borax)	/	3.1	/	13	/	/	/	/	/
Locknit PP Knit	218	1.9	109	54	26	13	0.0014	0.99	19.8
Interlock PP Knit	442	3.9	71	37	28	16	0.0009	0.99	9.3
Double Face PP Knit	690	6.1	72	45	29	19	0.0008	0.99	6.3
Interlock 2 Yarns PP Knit	698	6.2	80	42	26	16	0.001	0.99	11.6
Ottoman PP Knit	1195	10.6	100	59	37	16	0.0011	0.97	17.8
1 mm-Thick PP Sheet	1145	10.1	66	46	30	19	0.0008	0.99	5.3
2 mm-Thick PP Sheet	2048	18.1	86	55	38	21	0.0007	0.99	10.3
4 mm-Thick PP Sheet	3831	33.9	101	60	40	24	0.0006	0.99	9.7
6 mm-Thick PP Sheet	5729	50.6	131	68	39	23	0.0007	0.99	14.7
Balsa	/	1.2	10	5	3	2	0.0079	0.99	10.6
**Sample**	**Area Density (g/m^2^)**	**Sample Weight (g)**	**20 kW/m^2^**	**30 kW/m^2^**	**40 kW/m^2^**	**50 kW/m^2^**	**Slope (m^2^/(kW.s))**	**R^2^**	**CHF (kW/m^2^)**
Thin Aramid Fabric	/	2	/	7	5	/	0.0057	/	5
Thick Aramid Fabric	/	6.3	35	20	16	11.5	0.0019	0.99	4.6

**Table 4:** Main data for pHRR of thermally thin materials from cone calorimeter tests.

Sample	Area Density (g/m^2^)	Sample Weight (g)	25 kW/m^2^	35 kW/m^2^	50 kW/m^2^	75 kW/m^2^	Slope pHRR	EHC (kJ/g)	Fire Load (kJ)
Cotton Fabric (T1)	270	2.6	121	143	169	207	1.7	14.8	38.48
Cotton Fabric (T2)	210	2.2	109	123	140	157	0.94	13.6	29.92
Cotton Fabric (T3)	150	1.53	91	106	116	144	1.02	14.3	21.879
Cotton Fabric (T4)	180	1.78	97	103	124	150	1.1	12.4	22.072
Cotton Fabric (T5)	110	1.1	63	65	76	98	0.73	12.4	13.64
Cotton Fabric (T6)	170	1.53	72	89	111	141	1.37	12.2	18.666
Cotton Fabric (T7)	80	0.85	65	66	85	117	1.1	12.6	10.71
Cotton Fabric (T8)	210	2.08	92	99	123	170	1.61	12.8	26.624
FR T8 Cotton Fabric (F1)	250	2.46	85	113	135	186	1.96	10.8	26.568
FR T8 Cotton Fabric (F3)	250	2.45	49	103	103	139	1.54	8.8	21.56
Locknit Cotton Knit	200	2.9	67	91	88	156	1.67	12.2	35.38
Interlock Cotton Knit	400	3.84	85	100	125	181	1.93	11.3	43.392
Double Face Cotton Knit	500	4.46	98	110	128	195	1.95	11.8	52.628
Ottoman Cotton Knit	900	9.5	102	120	163	209	2.19	11.1	105.45
Flax Fabric	200	2.24	88	101	127	180	1.86	11.6	25.984
FR Flax Fabric	200	2.16	61	80	111	131	1.4	8.3	17.928
Locknit Flax Knit	700	5.7	153	178	196	299	2.87	12.9	73.53
Interlock Flax Knit	1100	10.6	158	198	227	301	2.76	12.4	131.44
Double Face Flax Knit	1100	10.1	164	186	210	291	2.52	12.9	130.29
Ottoman Flax Knit	1850	17.9	136	176	237	332	3.92	13.7	245.23
Jute Knit	380	3.95	126	141	189	245	2.46	10.8	42.66
Jute Knit (Rochelle Salt)	/	3.5	/	88	/	/	/	9.5	33.25
Jute Knit (Borax)	/	3.1	/	71	/	/	/	13.1	40.61
Locknit PP Knit	218	1.9	119	214	238	278	/	40	77.0499
Interlock PP Knit	442	3.9	215	281	369	576	7.2	40	156.22
Double Face PP Knit	690	6.1	320	348	480	710	8.1	40	243.874
Interlock 2 Yarns PP Knit	698	6.2	276	316	422	743	9.53	40	246.701
Ottoman PP Knit	1195	10.6	286	364	509	870	11.81	40	422.361
1 mm-Thick PP Sheet	1145	10.1	306	386	725	877	12.04	40	404.689
2 mm-Thick PP Sheet	2048	18.1	399	598	743	1093	13.4	40	723.845
4 mm-Thick PP Sheet	3831	33.9	607	746	1235	1764	24	40	1354.03
6 mm-Thick PP Sheet	5729	50.6	913	1252	1551	2100	23.1	40	2024.86
Balsa	/	1.2	110	124	136	200	1.78	15.6	18.72
**Sample**	**Area density (g/m^2^)**	**Sample weight (g)**	**20 kW/m^2^**	**30 kW/m^2^**	**40 kW/m^2^**	**50 kW/m^2^**	**Slope pHRR**	**EHC (kJ/g)**	**Fire load (kJ)**
Thin aramid fabric	/	2	/	78	114	/	/	13.9	27.8
Thick aramid fabric	/	6.3	99	131	148	185	2.75	13.8	86.94

## Data Availability

Not applicable.
